# Correlation between lipid accumulation product and epigenetic age acceleration in American adults: a cross-sectional analysis using NHANES data

**DOI:** 10.1186/s40001-024-02174-y

**Published:** 2024-12-03

**Authors:** Qiqiang Li

**Affiliations:** General Practice Department, Fuyong People’s Hospital of Baoan District, Shenzhen, 518103 China

**Keywords:** Epigenetic age acceleration, Lipid accumulation product, NHANES, Cross-sectional study, Horvathage

## Abstract

**Background:**

The risks of obesity and epigenetic age acceleration (EAA) have drawn widespread attention. Lipid accumulation product (LAP) is a simple and reliable indicator of obesity; however, the relationship between LAP and EAA remains unclear.

**Methods:**

Data from the National Health and Nutrition Examination Survey (NHANES) from 1999 to 2002 were used. The EAA was assessed using a self-administered questionnaire in the database. LAP was calculated based on triglycerides and waist circumference. The association between LAP and EAA was analyzed using logistic regression models, subgroup analysis, and smooth curve fitting.

**Results:**

A total of 1796 participants were included in the study, of whom 1055 had EAA. After adjusting for relevant covariates, participants with EAA generally had higher LAP levels than those without EAA (258.1 vs. 244.6). Logistic regression analysis showed that individuals in the highest LAP quartile (Q4) were more likely to have EAA than those in the lowest quartile (Q1) (OR = 1.77; 95% CI 1.31–2.39; *P* < 0.001). The area under the curve of the adjusted logistic regression analysis was 0.706.

**Conclusion:**

This research indicates that elevated LAP levels are independently linked to an increased risk of EAA, and early intervention to reduce high LAP levels is necessary to mitigate the progression of EAA.

**Supplementary Information:**

The online version contains supplementary material available at 10.1186/s40001-024-02174-y.

## Introduction

Epigenetic age acceleration (EAA) is a phenomenon in which an individual’s biological age exceeds their chronological age [1–7]. This concept is based on the epigenetic clock, which uses the methylation pattern of genes to estimate biological age, common biological clocks include HorvathAge, PhenoAge, GrimAgeMort, and others; for teenagers, the phenotype of HorvathAge is particularly prominent, with an accuracy of up to 98% [2, 3, 5, 7, 8]. It predicts an individual’s biological age by analyzing DNA methylation patterns, and epigenetic age often diverges from chronological age [1–9]. EAA has close and complex associations with cardiovascular diseases, neurodegenerative diseases, and others; factors such as sex, obesity, smoking, lack of exercise, dietary habits, and environmental exposure are closely related to EAA [9–28]. Studies have shown a strong correlation between obesity and EAA [14, 15, 28–31]; obesity and aging involve shared pathological mechanisms, including insulin resistance, with obesity capable of accelerating the aging process [14, 28], obesity is closely related to aging [27–29], with obesity being an independent influence on EAA [27–31].

LAP is a simple and easy-to-use index for assessing obesity. It combines triglycerides with waist circumference [32–36]; Compared to BMI and waist circumference, LAP shows better correlation in populations with visceral obesity [33, 34]; increased visceral fat is closely associated with cardiovascular and metabolic diseases, making LAP a more reliable index for evaluating visceral obesity in these contexts [33–35].

Previous studies have analyzed the relationship between obesity and EAA, previous studies used BMI as the measure of obesity, which evaluates overall obesity without distinguishing between subcutaneous and visceral fat obesity [27–31, 37]. However, research on visceral obesity and EAA is currently lacking. Therefore, this study utilized extensive data from the National Health and Nutrition Examination Survey (NHANES) to investigate the association between LAP and EAA.

## Materials and methods

### Survey description and study population

The research utilizes data from NHANES, a nationwide study by the National Center for Health Statistics (NCHS) that evaluates the nutritional and health status of U.S. adults and children. The NCHS Research Ethics Review Board approved all NHANES research protocols, ensuring that written informed consent was secured from all participants. Therefore, the NHANES database is freely available to researchers worldwide, and related studies do not require ethical approval or informed consent from participants. All details of the NHANES study design and data are publicly accessible at www.cdc.gov/nchs/NHANES/. This research followed the STROBE guidelines.

**Fig. 1:**
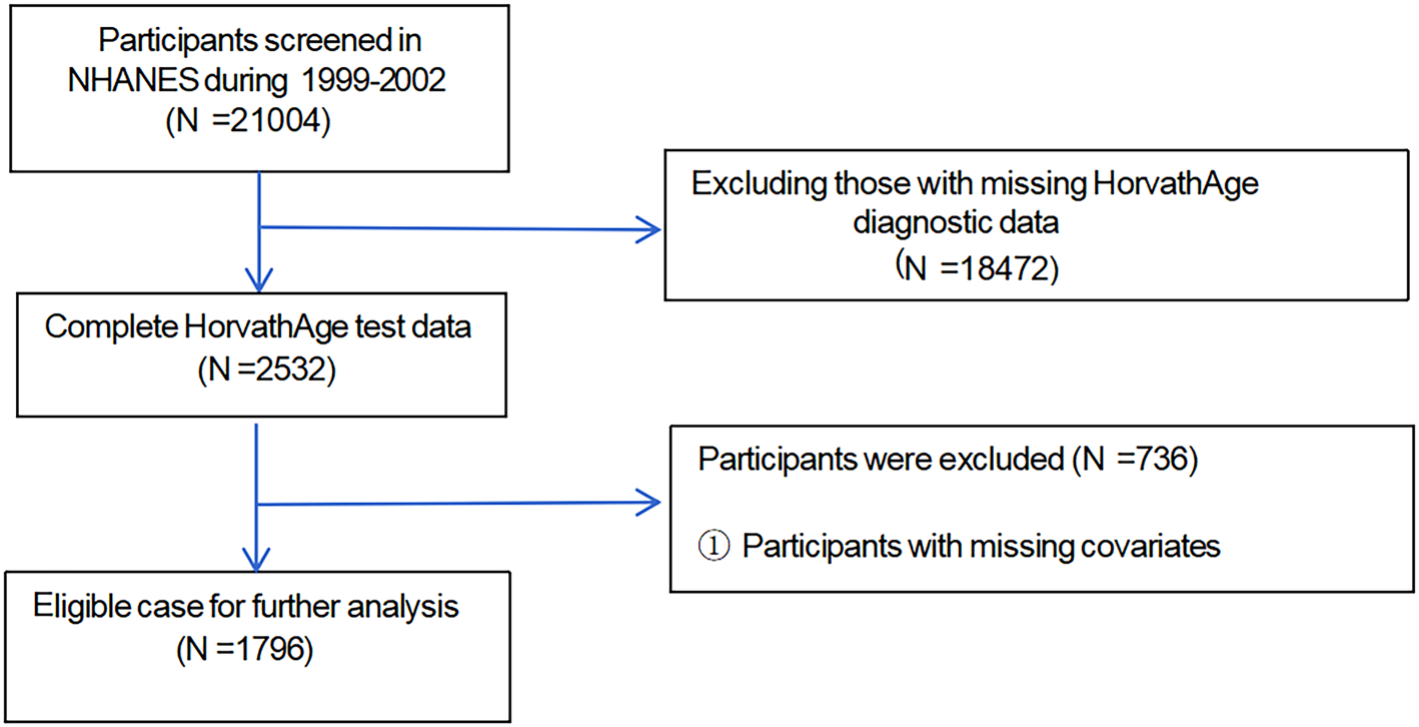
Flowchart of patients screening

Data from the NHANES 1999–2002 dataset were utilized in this study. Our analysis included participants with complete data on LAP and phenotypic age acceleration. The original sample included 21,004 participants. We excluded participants lacking data on phenotypic age acceleration diagnosis (*n* = 18,472) and those missing covariates such as hypertension diagnosis (*n* = 736), leaving a final sample of 1796 eligible participants (Fig. 1).

### Definition of epigenetic age acceleration and measurement of LAP

This study selected epigenetic age acceleration (EAA) as the outcome variable, where EAA is defined as the phenotypic age exceeding the chronological age. Specifically, participants were classified as EAA if their HorvathAge exceeded their chronological age, the relevant data are derived from the “DNA Methylation-Epigenetic Biomarkers (1999–2002)” file in the NHANES database.

LAP was selected as the exposure variable, calculated as ${\text{LAP}} = \left( {{\text{WC}} - {65}} \right) \times {\text{TG}}\;{\text{for}}\;{\text{males}}\;{\text{and}}\;{\text{LAP}} = \left( {{\text{WC}} - {58}} \right) \times {\text{TG}}\;{\text{for}}\;{\text{females}}.$

### Other clinical characteristics

To further evaluate the relationship between LAP and EAA, this study adjusted for 11 relevant covariates: age stratification, gender, hypertension, diabetes, smoking, alcohol consumption, race classification, marital status, exercise, education level, and family poverty ratio. All covariates are derived from the NHANES database and were referenced from the corresponding project descriptions [38–40].

### Statistical analysis

Data description and statistical analysis are based on the characteristics of the included sample. Continuous variables are usually expressed as mean (standard deviation, SD) or median (interquartile range, IQR), and categorical variables are indicated as counts (percentages), Differences between groups were compared using t-tests, Mann–Whitney U tests, and Chi-square tests. This study used logistic regression analysis to examine the risk relationship between LAP and EAA, constructing three logistic regression models with adjustments for different confounding factors. Model 1: no adjustment for confounding factors; Model 2: adjusted for age stratification, sex, and race; Model 3: further adjusted for age stratification, sex, alcohol consumption, race, education level, marital status, household income, hypertension, smoking status, diabetes, and active exercise. The VIF for the regression model was < 5, indicating that the conditions for applying logistic regression were met.

Finally, all analyses in this study were conducted using R version 4.3.3 (http://www.r-project.org). Robustness analysis was performed using stratified multivariable logistic regression models for subgroup analysis, with interaction analysis, stratified by factors such as age stratification, sex, smoking status, and marital status; propensity score matching was utilized to evaluate the sensitivity of the findings. Curve fitting was used to observe whether the relationship between LAP and epigenetic age acceleration was linear; if nonlinear, threshold analysis was conducted. Missing data were handled by direct deletion. Statistical significance was evaluated using two-sided values with *P* < 0.05. In statistical analysis, a p-value of less than 0.05 was considered to indicate a significant statistical association.

## Results

### Baseline characteristics

In the final analysis, a total of 1796 participants were included, with 1055 identified as having EAA. Table 1 provides the relevant baseline data. Notably, compared to the non-EAA group, the EAA group had a significantly lower age (62.3 vs. 70.4), higher LAP (73.8 vs. 65.2), and relatively higher proportions of smoking and diabetes (Table 1).

**Table 1:** Characteristics of participants by categories between LAP and EAA in NHANES

Characteristic	HorvathAge acceleration		*P*-value
	No (*N* = 741)	Yes (*N* = 1055)	
LAP, mean ± SD	65.2 ± 54.9	73.8 ± 59.5	0.002
Age, mean ± SD	70.4 ± 9.1	62.3 ± 8.9	< 0.001
BMI, mean ± SD	27.8 ± 4.9	29.1 ± 6.0	< 0.001
Sex (%)			< 0.001
Female	410 (55.3%)	456 (43.2%)	
Male	331 (44.7%)	599 (56.8%)	
Age (%)			< 0.001
50–59	85 (11.5%)	423 (40.1%)	
60–85	656 (88.5%)	632 (59.9%)	
Race (%)			0.836
Mexican American	205 (27.7%)	302 (28.6%)	
Other Hispanic	45 (6.1%)	63 (6%)	
Non-Hispanic white	312 (42.1%)	459 (43.5%)	
Non-Hispanic black	152 (20.5%)	199 (18.9%)	
Other races	27 (3.6%)	32 (3%)	
Education level (%)			0.215
Less than high school	333 (44.9%)	431 (40.9%)	
High school	153 (20.6%)	228 (21.6%)	
More than high school	255 (34.4%)	396 (37.5%)	
Marital status (%)			0.001
Married	441 (59.5%)	708 (67.1%)	
Others	300 (40.5%)	347 (32.9%)	
Poverty income ratio (%)			0.004
≤ 1.3 (%)	220 (29.7%)	279 (26.4%)	
> 1.3 and ≤ 3.5 (%)	304 (41%)	388 (36.8%)	
> 3.5 (%)	217 (29.3%)	388 (36.8%)	
BMI (%)			0.002
Normal weight	223 (30.1%)	253 (24%)	
Overweight	308 (41.6%)	429 (40.7%)	
Obesity	210 (28.3%)	373 (35.4%)	
Smoking status (%)			< 0.001
No	380 (51.3%)	448 (42.5%)	
Yes	361 (48.7%)	607 (57.5%)	
Alcohol consumption (%)			0.004
No	296 (39.9%)	351 (33.3%)	
Yes	445 (60.1%)	704 (66.7%)	
Hypertension (%)			0.210
No	378 (51%)	571 (54.1%)	
Yes	363 (49%)	484 (45.9%)	
Diabetes (%)			0.609
No	616 (83.1%)	866 (82.1%)	
Yes	125 (16.9%)	189 (17.9%)	
Exercise (%)			0.273
No	571 (77.1%)	837 (79.3%)	
Yes	170 (22.9%)	218 (20.7%)	
C-reactive protein (%)			0.205
Abnormal	371 (50.1%)	495 (46.9%)	
Normal	370 (49.9%)	560 (53.1%)	

### Multivariable analysis including LAP

In the multivariable analysis adjusted for relevant covariates, we created logistic regression models. This study observed that, compared with the first quartile (Q1) of LAP, participants in the fourth quartile (Q4) of LAP had an increased likelihood of EAA (OR = 1.77; 95% CI 1.31–2.39; *P* < 0.001). After including all covariates, the AUC for Model 3 was 0.706, indicating that Model 3 had good discrimination (Table 2).

**Table 2:** Association between LAP and EAA

Exposure	Model 1 [a]	Model 2 [b]	Model 3 [c]
	OR(95% CI) *P* value	OR(95% CI) *P* value	OR(95% CI) *P* value
LAP	1.17 (1.08, 1.27)	1.21 (1.11, 1.33)	1.19 (1.09, 1.31)
	< 0.001	< 0.001	< 0.001
LAP quartile			
Q1	Ref	Ref	Ref
Q2	1.11 (0.85, 1.44)	1.22 (0.92, 1.62)	1.22 (0.92, 1.63)
	0.451	0.161	0.161
Q3	1.16 (0.89, 1.51)	1.33 (1.00, 1.77)	1.30 (0.98, 1.74)
	0.268	0.050	0.070
Q4	1.67 (1.28, 2.19)	1.85 (1.38, 2.48)	1.77 (1.31, 2.39)
	< 0.001	< 0.001	< 0.001

Model 1<a>: adjusted for no covariates

Model 2<b>: adjusted for age stratification, race, sex

Model 3<c>: adjusted for sex, age stratification, race, poverty income ratio, education, marital status, smoking, alcohol use, hypertension, diabetes, exercise

### Optimization of the full covariate model and subgroup analyses and sensitivity analyses

For regression analysis with all covariates, we used the Change-in-Estimate method and Least Absolute Shrinkage and Selection Operator (LASSO) method to screen meaningful influencing factors. When an influencing factor was removed from the model and the effect value of the target factor changed by more than 10%, the factor was included. The selected multiple factors were: age stratification, sex, smoking, and marital status. The optimized regression model included five factors: age stratification, sex, smoking status, marital status, and LAP stratification. The results showed an AUC of 0.704 for the optimized model, and the Hosmer–Lemeshow test had a *P* > 0.05, indicating that the optimized model has good discrimination and calibration.

**Fig. 2:**
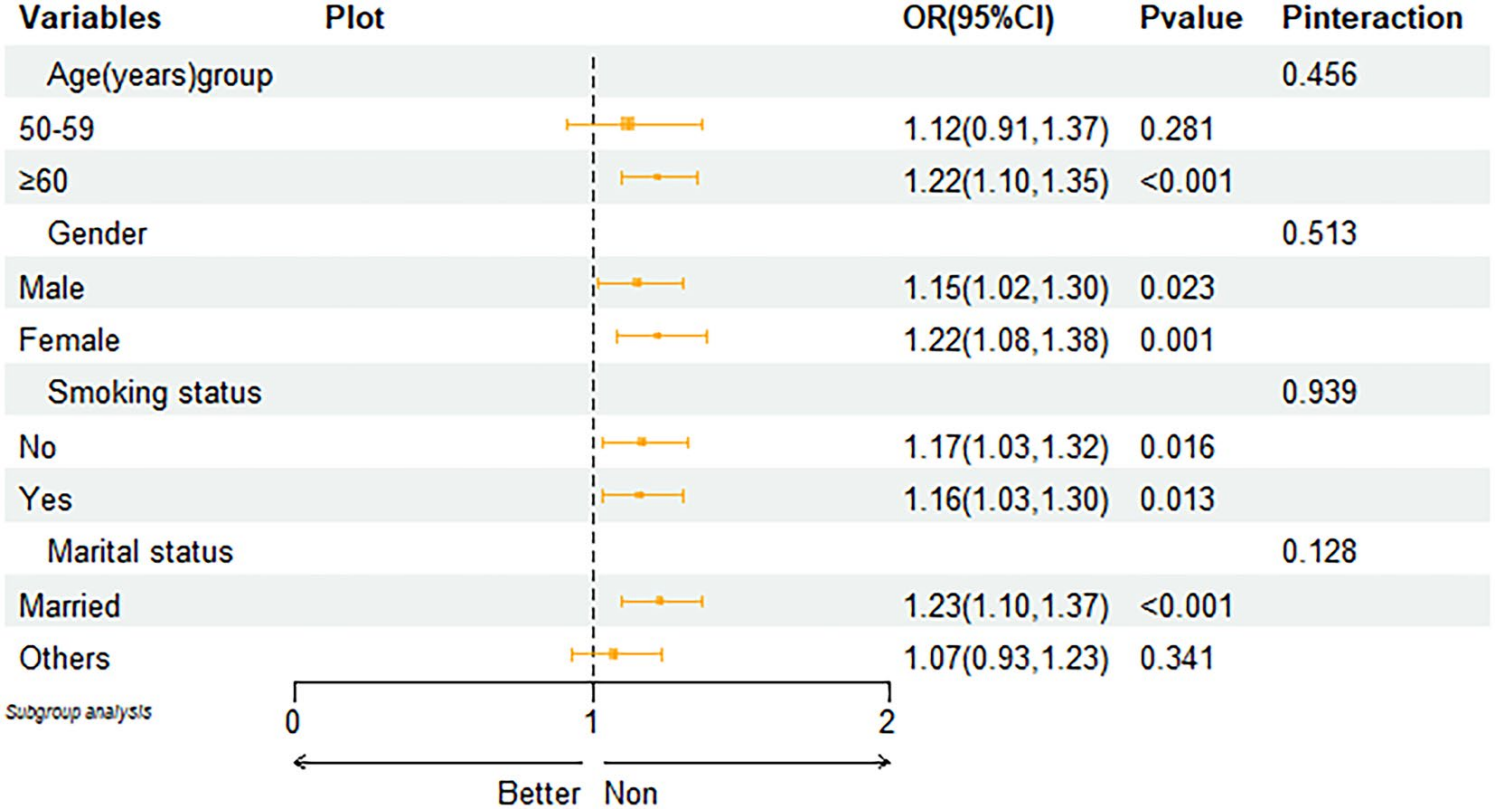
Subgroup analysis

In terms of robustness analysis, to evaluate the association between LAP and epigenetic age acceleration, we performed a stratified subgroup analysis. In subgroup analysis, after adjusting for relevant covariates, LAP remained closely associated with epigenetic age acceleration across different age stratification, sex, and smoking groups. Refer to Fig. 2 for specific details.

After adjusting for covariates, there was no mediating effect between age stratification, gender, smoking, marital status, and LAP.

**Fig. 3:**
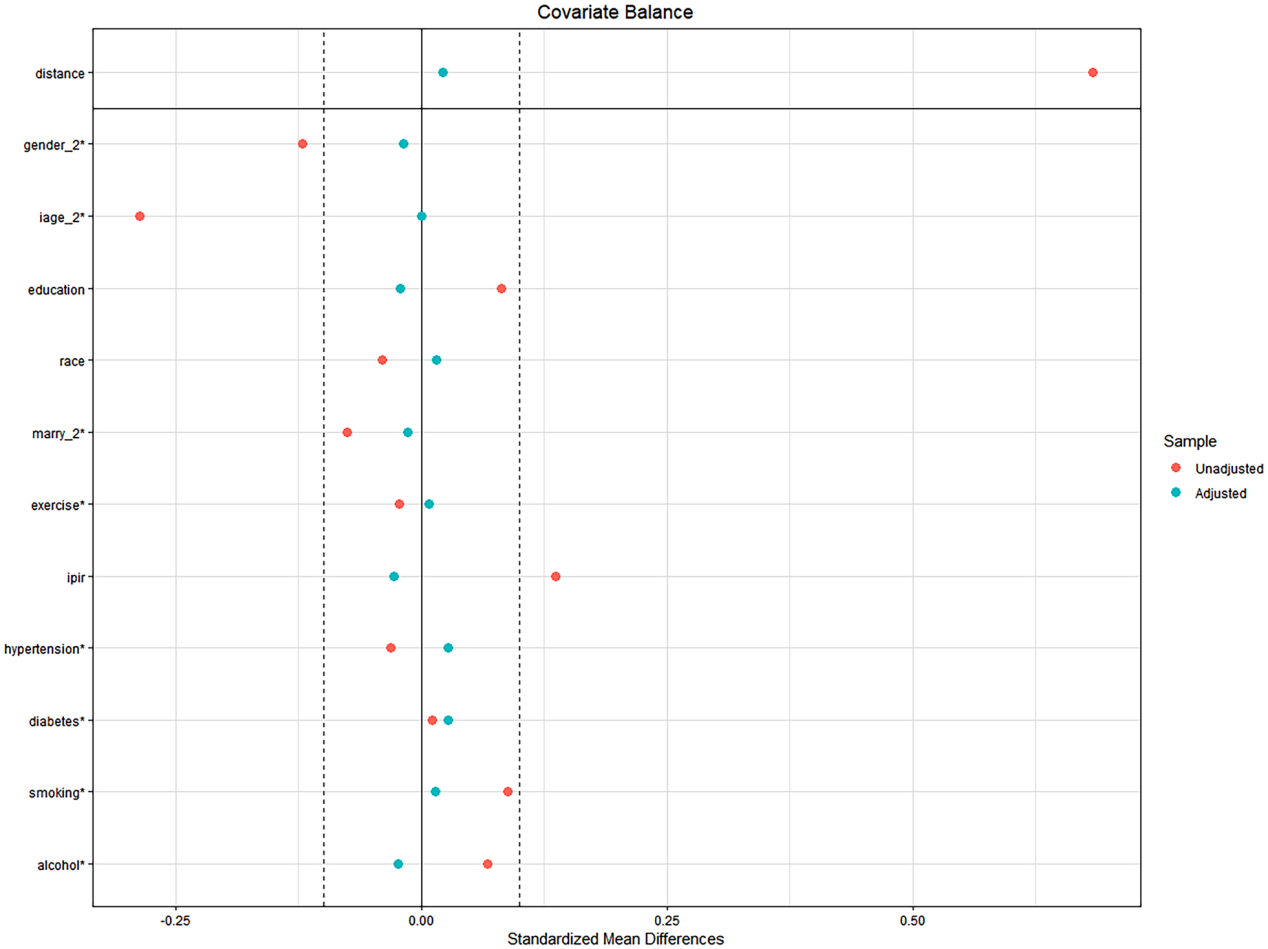
Propensity score matching

In sensitivity analysis, we also used the propensity score matching method. After matching for ten covariates, including age stratification, sex, and race, a sample of 1276 was obtained, with 638 participants each in the EAA and non-EAA groups. The matched plot is shown in Fig. 3. The *t*-test was used to examine LAP between the two groups, with *P* < 0.001. This indicates a significant statistical difference in LAP between the two groups and confirms that the association between LAP and EAA is robust.

### Curve fitting and threshold effects

Restricted cubic splines were used to analyze the relationship between LAP and EAA. After adjusting for relevant covariates, the relationship between the two is shown in Fig. 4 of supplementary materials. The figure shows nonlinear relationship (*P* = 0.034), and threshold effect analysis can be seen in Table 3.

**Table 3:** Threshold effect analysis

	OR	95%CI	*P*-value
LAP			
Inflection point	41.12		
< 41.12	0.96	0.81–1.12	0.579
≥ 41.12	1.11	0.99–1.24	0.070
Log likelihood ratio	< 0.001		

## Discussion

In this cross-sectional study involving 1796 samples, we found a significant positive correlation between LAP and EAA. Importantly, there is a nonlinear relationship between LAP and EAA (*P* = 0.034); the LAP inflection point is 41.12, with an OR of 0.96 before the inflection point and an OR of 1.11 after, showing a 15.6% increase in OR after the inflection point. After adjusting for relevant covariates, the regression results showed that higher LAP was associated with an increased likelihood of EAA; compared with the first quartile (Q1) of LAP, the fourth quartile (Q4) had a 77% increased risk of EAA, with an odds ratio of 1.77. There was a significant difference between the two, and the fully adjusted model had an AUC of 0.706. In terms of population attribution, the regression analysis of the adjusted model showed that the population attributable fraction was 41.6% for age stratification, 10.4% for LAP, 8.5% for gender, 4.0% for smoking, and 3.5% for marital status. These findings align with previous research, suggesting that LAP is an independent influencing factor for EAA [27–31, 41].

In the subgroup analysis, we found that the correlation between LAP and EAA was stronger in people over 60 years old compared to those under 60, with an OR increase of 10% and *P* < 0.001. In terms of gender, females had a higher OR than males, with a 7% increased probability of EAA in females compared to males.

Obesity is a major global public health issue, and LAP is a reliable, simple, and effective indicator of obesity [14, 15, 27–36]. EAA represents the biological aging of the human body, with DNA methylation playing a crucial role. EAA is closely associated with tumors, endocrine metabolism, cardiovascular diseases, etc., and HorvathAge is a key indicator for evaluating EAA [6–15]. There is a close relationship between obesity and EAA, as obesity accelerates biological aging [19–22, 27–31]. This study marks the first exploration of the relationship between LAP and EAA, specifically in relation to HorvathAge.

However, the potential mechanisms between LAP and EAA remain to be studied, and the following explanations are possible. LAP is a reliable indicator of obesity and is closely related to visceral obesity [32–36]. In obese populations, the expansion of white adipose tissue and the resulting local hypoxia lead to abnormal secretion functions in adipose tissue. Inflammatory-related adipokines are abnormally secreted, activating immune cells (particularly monocytes and lymphocytes). The release of pro-inflammatory cytokines mediated by immune cells interferes with insulin signaling pathways, causing insulin resistance, which then affects blood glucose and the endocrine system. The abnormal secretion of adipokines causes low-grade chronic inflammation, stimulating the formation of TNF-α and IL-6, which inhibits the action of adiponectin, affecting insulin secretion, anti-inflammatory responses, and cardiac function [23–29]. Additionally, the decline in insulin receptors on hypertrophic adipocytes reduces insulin effectiveness, while the increase in peripheral free fatty acids in obese individuals can damage peripheral tissues and decrease insulin utilization. All of these factors contribute to increased production of reactive oxygen species, leading to oxidative stress [23–29, 41–43].

Oxidative stress is a major pathogenic factor in obesity-related comorbidities, causing insulin resistance in peripheral tissues and is closely related to endocrine diseases such as diabetes. Prolonged oxidative stress can damage the blood–brain barrier, accelerating neurodegenerative changes in the brain. Oxidative stress impairs cardiovascular function, leading to an increased incidence of cardiovascular disease. It also damages mitochondrial function, resulting in a range of diseases, particularly tumors [28, 29].

Insulin resistance, low-grade chronic inflammation, and impaired immune function are characteristics of obesity as well as aging, which is particularly evident in the elderly [25–31]. In the subgroup analysis of this study, the OR for EAA increased by 10% in elderly individuals over 60 compared to those under 60, which further supports this conclusion. Various measures to reduce the lipid accumulation product (LAP) can improve epigenetic age acceleration (EAA), studies have shown that weight loss, smoking cessation, and a healthy diet can effectively reduce EAA [2, 44].

### Advantages and limitations

Specifically, this study has the following advantages. First, our research is based on NHANES data, which offers the advantage of a large sample size. In addition, we adjusted for relevant covariates and conducted subgroup analysis and propensity score matching, making the results of this study robust and reliable.

However, this study still has some limitations. Firstly, although we adjusted for covariates as thoroughly as possible, there may be uncontrolled factors; however, the model’s AUC after adjusting for all covariates was 0.706, indicating that our study still has good clinical significance. Secondly, our study population was limited to American adults over 50. Therefore, additional studies are required to assess if these findings can be extrapolated to other nations.

## Conclusion

This study provides a new clinical approach. By assessing several key risk factors—age stratification, sex, LAP stratification, smoking, and marital status—the probability of epigenetic age acceleration in American adults can be conveniently estimated, with a differentiation rate of 70.6%. This helps clinicians quickly identify epigenetic age acceleration in middle-aged and older adults, enabling targeted care and early intervention to improve patient outcomes.

This study indicates that higher LAP is associated with an increased risk of EAA, he RCS analysis demonstrates that there is a nonlinear relationship between LAP and EAA risk, as well as a linear relationship with EAA risk. These findings suggest that LAP, as a concise indicator of visceral obesity, shows beneficial and positive value in clinical practice, aiding clinicians in making professional and reasonable recommendations. This study highlights that early intervention to reduce LAP levels is essential to slow the progression of EAA in American adults.

## Supplementary Information

Supplementary Material 1.

Supplementary Material 2.

Supplementary Material 3.

## Data Availability

The original data presented in this research are available within the article and its supplementary material. For further inquiries, please reach out to the corresponding author.

## Abbreviations

LAP: Lipid accumulation product
EAA: Epigenetic age acceleration
NHANES: National Health and Nutrition Examination Survey
NCHS: National Center for Health Statistics
SD: Standard deviation
IQR: Interquartile range
LASSO: Least Absolute Shrinkage and Selection Operator
