# Supplementary material for: Correlation between lipid accumulation product and epigenetic age acceleration in American adults: a cross-sectional analysis using NHANES data
Source: Eur J Med Res. 2024 Dec 3;29:575. doi: 10.1186/s40001-024-02174-y (PMC11613462; doi:10.1186/s40001-024-02174-y)

Supplementary Material

**（1）Data Sources and Statement----------------------------------------------------------Page 2**

**（2）Ethics statement--------------------------------------------------------------------------Page 3**

1. **Author Information--------------------------------------------------------------------- Page 4**
2. **Figure and Table------------------------------------------------------------------------- Page 5**

**Data Sources and statement**

1.1 Source of Original Data

All data were sourced from the NHANES database, which is available for download from the official NHANES website.

NHANES website at http://www.cdc.gov/nchs/NHANES/.

1.2 Integration of Original Data

Data on HorvathAge were available only for the 1999-2002 periods. After downloading, the data were integrated and summarized based on the SEQN (sequence number).

1.3 Explanation of Data Codes

Data were extracted from NHANES, and the explanations for each parameter are as follows

| Variables | NHANES Code | Explanation |
| --- | --- | --- |
| SEQN | SEQN |  |
| LAP |  | lipid accumulation product |
| gender | RIAGENDER |  |
| age | RIDAGEYR |  |
| diabetes | DIQ010 |  |
| race | RIDRETH1 |  |
| Education level | DMDEDUC2 |  |
| marry | DMDMARTL |  |
| pir | INDFMPIR | Poverty income ratio |
| hypertension | BPQ020 |  |
| Smoking status | SMQ020 |  |
| Alcohol status | ALQ101 |  |
| bmi status | BMXBMI |  |
| HorvathAge | HorvathAge | DNA Methylation-Epigenetic Biomarkers (1999-2002) |
| Exercise | PAD020,PAQ100,PAQ180 |  |
| C-Reaction Protein | LBXCRP, LB2CRP |  |
| lap4 |  | Quartile Classification of LAP |

1.4 Handling of Missing Values

Missing values were handled by direct deletion. Any row with missing values for the relevant parameters was removed from the dataset.

1.5 Handling of Outliers

Since logistic regression was employed in the analysis, it is relatively insensitive to outliers. Therefore, data points beyond three standard deviations from the mean were retained in the dataset.

1.6 Parameter Classification and Encoding

Parameters were categorized and encoded, typically setting 0 or 1 as dummy variables. The main classifications are as follows

| Parameter | Classification Encoding |
| --- | --- |
| iage | 50~59=1,60~85=2 |
| diabetes | Yes=1, No=0 |
| hypertension | Yes=1, No=0 |
| Smoking status | Yes=1, No=0 |
| Alcohol status | Yes=1, No=0 |
| Marital status | Married=1, others=2 |
| Education level | Less than high school=1,High school=2,More than high school=3 |
| ipir | (Poverty income ratio)≤1.3 =1,(Poverty income ratio)>1.3 and ≤3.5 =2,  (Poverty income ratio)>3.5 =3 |
| race | Non-Hispanic white=1,Non-Hispanic black=2,Mexican American=3,  Other races=4,Other Hispanic=5 |
| Epigenetic Age Acceleration | Yes=1, No=0 |
| BMI status | BMI<25, 25≤BMI<30, BMI≥30 mapping 1, 2, 3 |
| lap4 | To LAP, lap4<33.9, 33.9≤ieg4 <55.1, 55.1≤ieg4<88.1, ieg4≥88.1 mapping 1, 2, 3, 4 |
| exercise | Yes=1, No=0 |
| C-Reaction Protein | ≥3，<3 mapping 1,0 |
|  |  |

1.7 Regression Analysis Method

Logistic regression method was used for the analysis, and VIF <10.

1.8 Upload of original Data

The original data that has been integrated will be uploaded as an attachment to the supplementary materials.

**Ethics**

2.1 Ethics Statement

The National Center for Health Statistics (NCHS) research ethics review board (ERB) approved the NHANES study protocol, and participants provided written informed consent at enrollment. The NCHS Institutional Review Board/ethics review board (IRB/ERB) protocol numbers of 1999–2004 National Health and Nutrition Survey is ‘#98-12’.

(the website is https://www.cdc.gov/nchs/nhanes/irba98.htm).

We have carefully examined our study in light of the Ethical Review Methods for Life Science and Medical Research Involving Human Beings. We have found that Article 32 of this regulation specifically exempts research from requiring ethical approval under certain conditions.

According to Article 32: "Ethical approval is not required for research that meets the criteria of (a) using legally obtained public data, or data generated by observation and not interfering with public behavior; and (b) using anonymized informational data to conduct the research."

Since this study was based on publicly available deidentified data and informed consent was waived, ethical approval and consent were not required.

This research used publicly available data from the National Health and Nutrition Examination Survey (NHANES), with all details accessible on the official website <https://www.cdc.gov/nchs/nhanes>.

**Author Information**

3.1 Author Contributions

All authors contributed significantly to the research and preparation of this manuscript. Specific contributions are as follows:

Author QL: Conceptualization, data curation, formal analysis, writing—original draft, writing—review & editing.

All authors have read and approved the final manuscript.

3.2 The corresponding author and contact information

Correspondence: Qiqiang Li

1. mail: [41439608@qq.com](mailto:41439608@qq.com)

3.3 Conflict of interest

The authors state that the research was conducted without any potential conflict of interest.

3.4 Data Availability Statement

The original data presented in this research are available within the article and its supplementary material. For further inquiries, please reach out to the corresponding author.

**Figure and Table**

4.1 Fig 1 Flow chart of patients screening


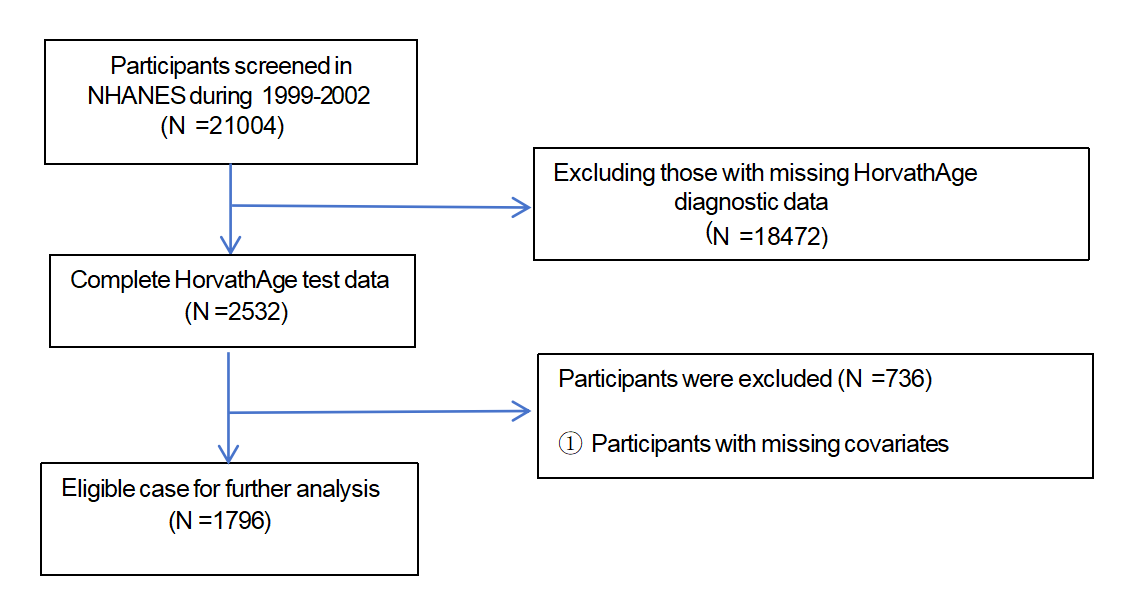


4.2 Fig 2 Subgroup analysis


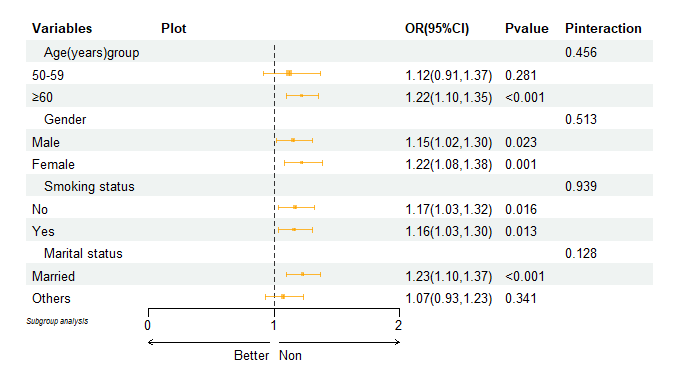


4.3 Fig 3 propensity score matching


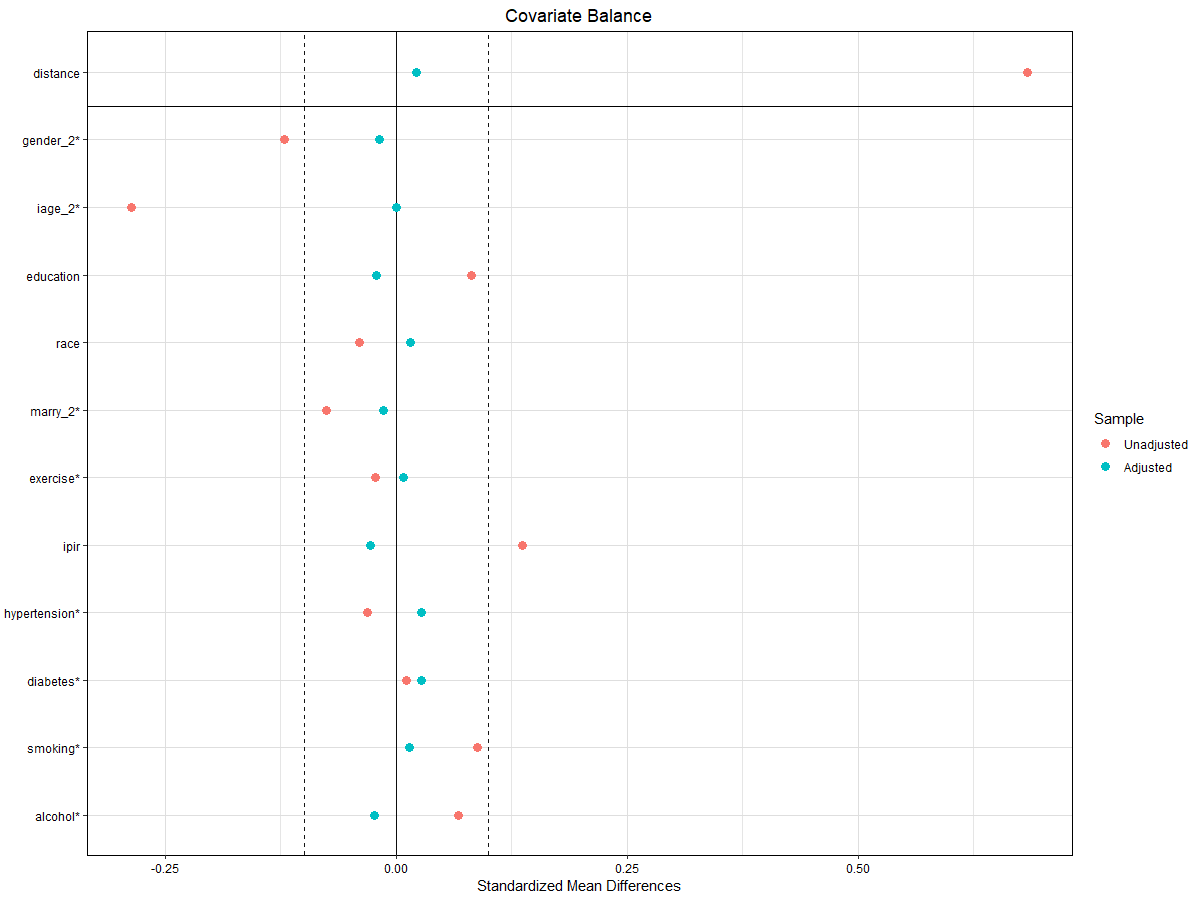


4.4 Fig 4 Restricted cubic splines


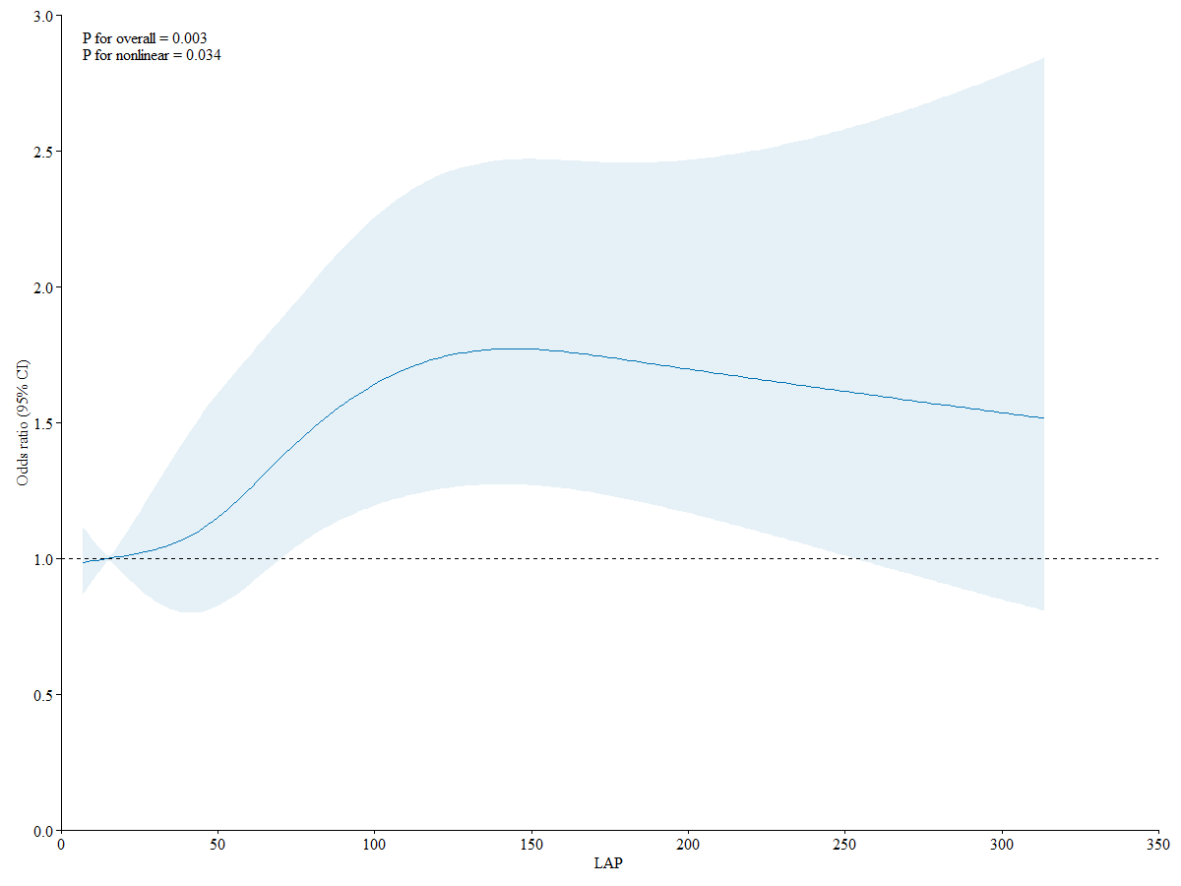


4.5 Fig 5 Variable selection based on the LASSO method


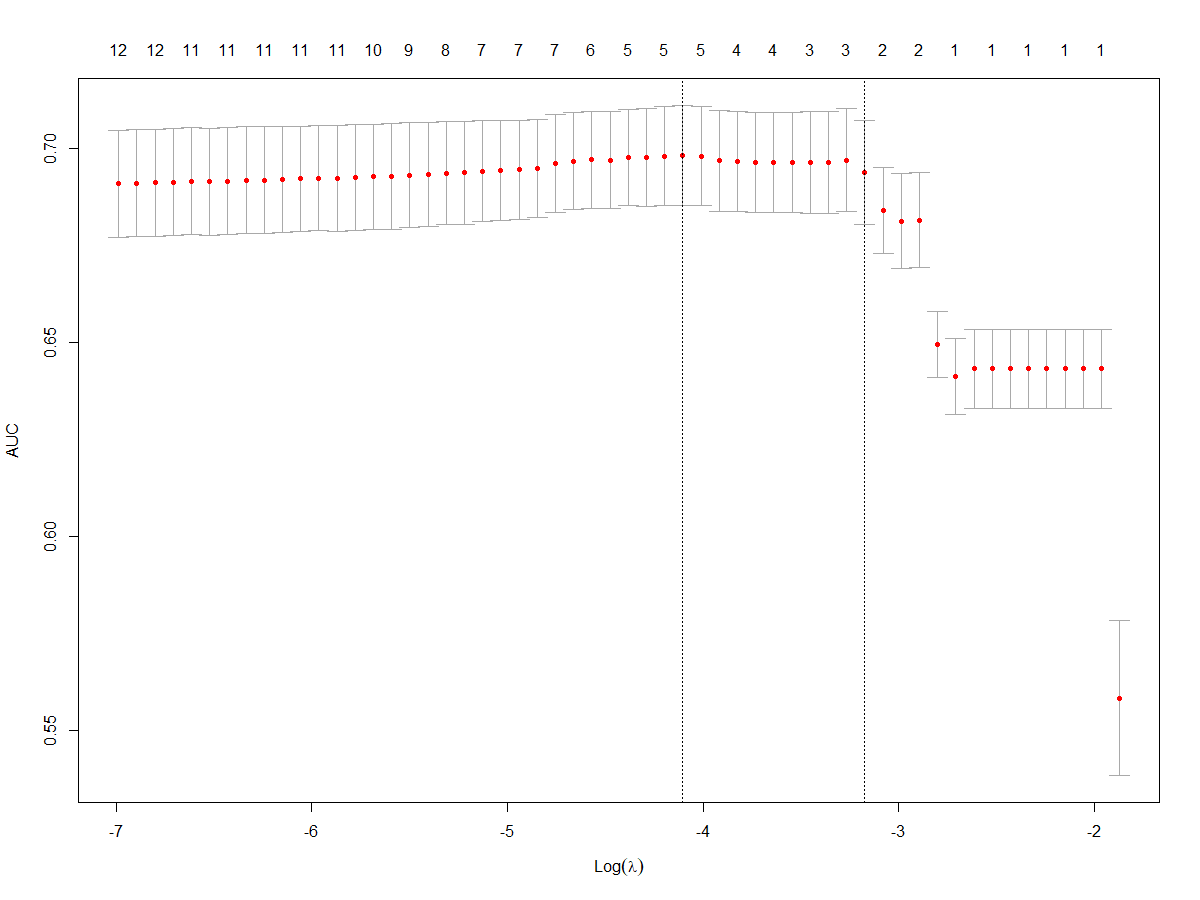

Supplement: Supplementary file 1 — Supplementary Material 1. [file 40001_2024_2174_MOESM1_ESM.docx]
